# Decoding immune‐driven erythroid failure in pure red cell aplasia

**DOI:** 10.1111/bjh.70473

**Published:** 2026-04-06

**Authors:** Federico Spataro, Vanessa Desantis, Antonio Giovanni Solimando

**Affiliations:** ^1^ Department of Precision and Regenerative Medicine and Ionian Area ‐ DiMePRe‐J, Section of Pharmacology University of Bari Aldo Moro Bari Italy; ^2^ Department of Precision and Regenerative Medicine and Ionian Area ‐ DiMePRe‐J, Guido Baccelli Unit of Internal Medicine, School of Medicine University of Bari Aldo Moro Bari Italy

**Keywords:** pure red cell aplasia, STAT3, T‐cell clonality

## Abstract

Pure red cell aplasia (PRCA) is increasingly recognised as a T‐cell‐mediated bone marrow failure syndrome, yet its immunogenetic drivers remain poorly defined. In their paper, Yamashita et al. integrate human leucocyte antigen (HLA) typing, T‐cell receptor repertoire analysis and mutational profiling to reveal enriched HLA alleles, *signal transducer and activator of transcription 3* (*STAT3*)‐mutated T‐cell clones and a shared T cell receptor beta (TCRβ) motif in PRCA patients. These findings suggest that antigen‐driven cytotoxic T‐cell responses represent a central mechanism underlying erythroid suppression.

Commentary on: Yamashita et al. Immunological features of acquired pure red cell aplasia: Specific human leucocyte antigen alleles, signal transducer and activator of transcription 3 mutations and a unique T‐ cell receptor beta motif. Br J Haematol 2026; 208:1797–1805.

In their paper, Yamashita et al. investigate the immunogenetic architecture of acquired pure red cell aplasia (PRCA) by integrating human leucocyte antigen (HLA) typing, targeted mutational analysis and T‐cell receptor repertoire profiling.^1^ Their work identifies specific HLA alleles enriched in PRCA patients and links these genetic backgrounds to clonal cytotoxic T‐cell expansions carrying *STAT3* mutations and shared TCRβ motifs.

PRCA is defined by severe anaemia with reticulocytopenia and virtually no erythroid precursors in the marrow.^2^, ^3^ It is often linked to autoimmune conditions or thymoma, but idiopathic cases are known to be immune‐mediated. Classical work showed that CD8^+^ T cells from a PRCA patient could suppress erythropoiesis only in HLA‐matched marrow, implying a specific T‐cell attack.^4^ Yamashita et al. build on this concept by providing a comprehensive immunogenetic analysis of PRCA in a cohort of 39 Japanese patients.

One of the most striking observations reported by Yamashita et al. is the enrichment of specific HLA alleles in PRCA patients. In particular, HLA‐B*44:03:01, HLA‐C*14:03 and HLA‐DRB1*13:02 were significantly overrepresented compared with population reference datasets. These findings suggest that antigen presentation may shape pathogenic T‐cell responses in PRCA, providing a potential immunogenetic framework for disease susceptibility. In line with this interpretation, an extended haplotype including A*33:03–C*14:03–B*44:03:01–DRB1*13:02 was also enriched among patients.

The authors further demonstrate that the majority of PRCA patients display mono‐ or oligoclonal expansions of cytotoxic T cells, indicating a restricted T‐cell receptor repertoire consistent with antigen‐driven immune activation. Indeed, approximately 80% of patients exhibited dominant CD8^+^ T‐cell clones with markedly reduced repertoire diversity. Such findings reinforce the concept that PRCA belongs to the broader spectrum of immune‐mediated cytopenias driven by clonal T‐cell responses. Accumulating evidence indicates that cytotoxic CD8^+^ T cells play a central role in suppressing erythropoiesis through antigen‐driven immune responses and clonal T‐cell expansions (Figure 1). Similar immune circuits have been described across the spectrum of immune‐mediated bone marrow failure syndromes, including T‐cell large granular lymphocytic leukaemia and other immune cytopenias.^5^, ^6^

**FIGURE 1:**
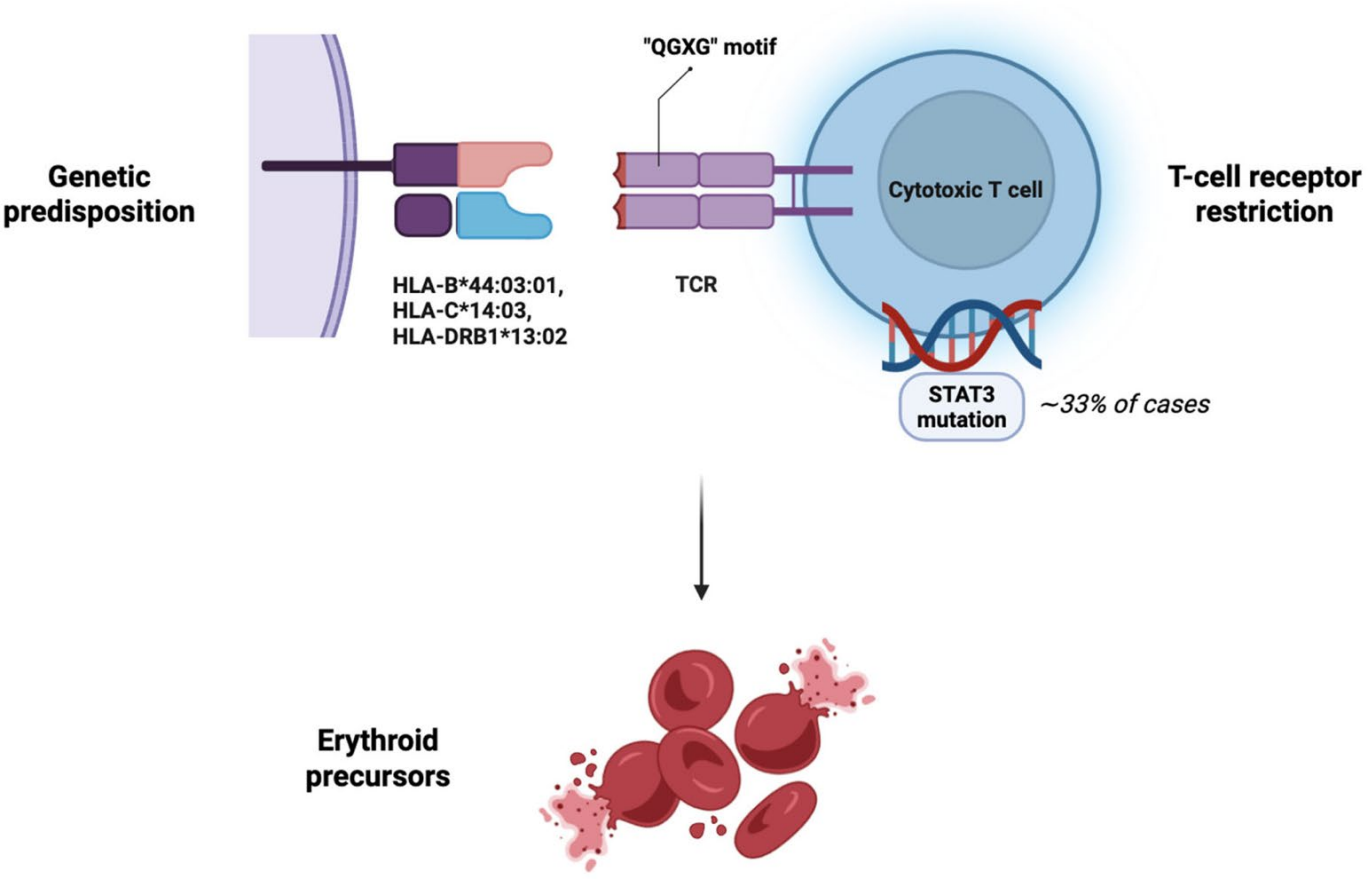
The immunogenetic axis of acquired pure red cell aplasia. Predisposing human leucocyte antigen (HLA) alleles present antigenic peptides to T cells, promoting activation of cytotoxic CD8^+^ T‐cell responses. Antigen‐driven stimulation leads to clonal expansion and restriction of the T‐cell receptor repertoire, with recurrent TCR motifs (e.g. ‘QGXG’: Q, glutamine; G: glycine; X: any amino acid) suggesting convergent antigen recognition. A subset of expanded clones acquires activating *STAT3* mutations, enhancing survival, proliferation and resistance to apoptosis. These persistent cytotoxic T‐cell clones mediate immune suppression of erythropoiesis through direct or cytokine‐mediated effects on erythroid progenitors, ultimately resulting in selective depletion of erythroid precursors in the bone marrow. [Colour figure can be viewed at wileyonlinelibrary.com]

Importantly, *STAT3* mutations were detected in approximately one‐third of cases, linking PRCA to the biological landscape of cytotoxic T‐cell disorders such as T‐cell large granular lymphocytic leukaemia. STAT3 activation may promote survival and expansion of pathogenic T‐cell clones, thereby sustaining immune‐mediated erythroid suppression.^7^, ^8^ Notably, Yamashita et al. also identified a recurrent ‘QGXG’ motif within the complementar‐determining region 3 beta (CDR3β) of the TCR in several expanded clones, suggesting convergent antigen recognition. These shared TCR motifs strongly support the hypothesis that antigen‐driven immune responses contribute to the pathogenesis of PRCA. Similar public TCR signatures have been described in viral antigen‐driven immune responses.^9^

These observations also fit within a broader framework of dysregulated Janus kinase‐signal transducer and activator of transcription (JAK–STAT) signalling in immune‐mediated bone marrow failure. Experimental and translational studies have shown that abnormal activation of STAT pathways, including signal transducer and activator of transcription 1 (STAT1) hyperactivation in bone marrow cells, may contribute to cytotoxic T‐cell–driven suppression of haematopoiesis in conditions, particularly in aplastic anaemia. From a therapeutic perspective, these findings further support the exploration of JAK–STAT targeting strategies in immune‐mediated marrow failure, raising the possibility that modulation of this pathway may have therapeutic relevance across immune cytopenias.^10^

Despite these important insights, several questions remain. The cohort size remains relatively limited and largely restricted to a single ethnic population, which may influence HLA allele distribution. Moreover, although shared TCR motifs strongly suggest antigen‐driven responses, the specific target antigens responsible for erythroid suppression remain to be identified.

Future studies integrating single‐cell transcriptomics, spatial bone marrow profiling and antigen discovery approaches will be required to identify the molecular targets driving these clonal T‐cell responses. These efforts may ultimately open the door to targeted immune‐modulating strategies in PRCA.

Together, the findings reported by Yamashita et al. place PRCA within a defined immunogenetic framework linking HLA background, *STAT3*‐driven T‐cell clones and restricted TCR repertoires. This work strengthens the concept of PRCA as an antigen‐driven immune bone marrow failure syndrome.

## AUTHOR CONTRIBUTIONS

**Federico Spataro:** Conceptualization; investigation; writing – original draft; methodology; writing – review and editing; software; visualization. **Vanessa Desantis:** Conceptualization; investigation; writing – review and editing; project administration. **Antonio Giovanni Solimando:** Conceptualization; investigation; funding acquisition; writing – original draft; methodology; writing – review and editing; supervision; visualization; validation; formal analysis; resources; data curation.

## FUNDING INFORMATION

This study was funded by the Italian network of excellence for advanced diagnosis INNOVA, ‘Ministero della Salute’ (code PNC‐E3‐2022‐23683266 PNC‐HLS‐DA, to VD and AGS) and by the European Union‐Next Generation EU‐PNRR M6C2—‘Investimento 2.1 Potenziamento e rafforzamento della ricerca biomedica nel SSN’ (project n. PNRR‐POC‐2022‐12375862 FUSION‐TARGET) to AGS. Moreover, this study was funded by ‘Fondo per il Programma Nazionale di Ricerca e Progetti di Rilevante Interesse Nazionale ‐ PRIN’ (code 2022ZKKWLW to AGS) and from the ‘Società Italiana di Medicina Interna‐SIMI’ 2023 Research Award (Camel to AGS).

## CONFLICT OF INTEREST STATEMENT

The authors declare no conflict of interest.

## Data Availability

The data that support the findings of this study are available from the corresponding author upon reasonable request.
